# Correlation between disability, internalized stigma and mental recovery in patients with bipolar disorder

**DOI:** 10.3389/fpsyg.2025.1396545

**Published:** 2025-04-04

**Authors:** Elif Nur Yıldız, Bedia Tarsuslu, Gulgun Durat

**Affiliations:** ^1^Department of Emergency, Kocaali District State Hospital, Sakarya Provincial Health Directorate, Sakarya, Türkiye; ^2^Department of Psychiatric Nursing, Faculty of Health Sciences, Ordu University, Ordu, Türkiye; ^3^Department of Psychiatric Nursing, Faculty of Health Sciences, Sakarya University, Sakarya, Türkiye

**Keywords:** bipolar disorder, internalized stigma, mental recovery, stigma, recovery, disability

## Abstract

**Background:**

This study aimed to examine the correlation between disability, internalized stigma and mental recovery in patients with bipolar disorder. It further examined the impact of internalized stigma and disability on mental recovery.

**Methods:**

The study was conducted with 103 patients diagnosed with bipolar disorder in remission who had been referred to a Community Mental Health Center. Data were collected using the Short Disability Assessment Schedule, Internalized Stigma of Mental Illness Scale and Recovery Process Inventory. Data were analyzed using an independent *t*-test, a one-way analysis of variance, and a multiple linear regression analysis.

**Results:**

No disability was diagnosed in 33.0% of the participants, while 25.2% had mild disability, 30.1% had moderate disability and 11.7% had severe disability. DAS-S scores indicated differences between the recovery process and internalized stigma scores (*p* < 0.05). While disability levels, alienation and perceived discrimination were not found to be effective on recovery, endorsement of stereotypes, social withdrawal, and resistance to stigma were found to be effective (F:43.343, *p* < 0.001, R^2^: 0.787).

**Conclusion:**

The prevalence of disability in patients with bipolar disorder increases the likelihood that these individuals will need more help from others. The mental recovery is positively affected by the endorsement of stereotypes, social withdrawal, and resistance to stigma.

## 1 Introduction

The incidence of bipolar disorder (BD) is 1%–3.7% of the general population worldwide. The World Health Organization (WHO) has reported that it is among the top 10 disorders leading to disability (Fiorillo et al., 2013; Yılmaz, 2020). The BD is a chronic mental disorder consisting of two subtypes. Bipolar I requires at least one manic or mixed episode throughout life, while Bipolar II is characterized by at least one hypomanic and recurrent depressive episode (American Psychiatric Association, 2013).

Problems in physical function can be considered disabilities when there is inhibition of movements and the capacity to participate in daily life, leading to feelings of inadequacy (Kesioğlu et al., 2003; Softa and Karaahmetoğlu, 2016). Disability in mental disorders is defined as the obstruction of an individual’s daily life due to impairments in their affective and cognitive functions due to mental disorders (Passerieux et al., 2018). BD ranks fourth among the diseases that cause disability in adolescents between the ages of 10 and 24 (Burdick et al., 2019). Disability in social functions is observed mainly during periods of depression. Factors such as genetic characteristics, the severity of the disease, periods of crisis and stress and anxiety determine the severity of the psychosocial disability of patients with BD (Vlad et al., 2018).

As a chronic and lifelong disorder, bipolar disorder carries significant risks in terms of social stigmatization and self-stigmatization (Huggett et al., 2018; Perich et al., 2022). This disorder may result in disability by causing loss of functionality and a decrease in quality of life. Public stigmatization occurs when individuals are stereotyped and discriminated against by those around them. This process can evolve into public stigma, where individuals are directly exposed to stigmatizing behaviors, and then into internalized stigma, where individuals accept and internalize these negative perceptions (Perich et al., 2022). However, it is important to note that not all individuals experience the same level of stigmatization, as personal, social and contextual factors shape these experiences.

Individuals with BD may experience stigmatization in different settings such as family, work, social environment and health institutions (Arguvanlı, 2018; Hawke et al., 2013). These stigmatization experiences may lead to feelings of shame, concealment of symptoms and even withdrawal from treatment. However, stigmatization differs from individual to individual, and not all individuals experience such affects similarly. Internalized stigma can negatively affect the mental recovery process by causing individuals to stigmatize themselves, develop negative self-perceptions and damage their self-esteem (Chang et al., 2016; Kök and Demir, 2017). In other words, most individuals with BD may see themselves as dangerous or internalize thoughts such as “I cannot manage my own life” (Çam and Çuhadar, 2011; Yanos et al., 2011).

Üstündaǧ and Kesebir (2013) concluded that the rate of internalized stigma in bipolar patients was 46%. Internalized stigma in BD may result in decreased functionality, low self-esteem, and deterioration of social relationships and quality of life (Post et al., 2018). It may also negatively affect treatment compliance and desire to seek treatment (Mittal et al., 2012). Studies conducted with the patients with BD revealed that internalized stigma may have an impact on social functioning; however, individuals who have experienced internalized stigma often have severe health conditions and are likely to be or previously have been hospitalized. It is not that clear whether internalized stigma affects disability or whether disability affects internalized stigma (Aydemir, 2013; Ellison et al., 2013). As a result of experiencing internalized stigma, the individual may withdraw from social life and suffer impaired functionality and alienation (de Filippis et al., 2022). However, internalized stigma and self-stigma are not the same concepts and work through different mechanisms. While internalized stigma means that the individual adopts social prejudices and develops negative judgments toward himself/herself, self-stigma is generally associated with decreased self-esteem and self-worth deterioration based on the individual’s internal processes. These two concepts address the affects of public stigmatization on the individual from different angles. It should not be forgotten that public stigmatization can shape the level of self-stigmatization and the experience of internalized stigmatization (Öztürk et al., 2021). In this context, the specific role of self-stigma in these processes should be examined and how internalized stigma affects the individual’s psychosocial wellbeing.

Recovery is traditionally defined as reducing of one’s symptoms of disorders and regaining one’s former lifestyle (Mueser et al., 2006). Mental recovery is not just about addressing the symptoms of illness. Cognitive and psychological factors and social relationships must also be evaluated and addressed (Lahera et al., 2018). Definitions of the concept of recovery commonly include a sense of hope, being able to determine one’s future, having a stronger sense of the self and one’s positive characteristics and being capable of living a meaningful life while managing its negative aspects (Australian Health Ministers’ Advisory Council, 2013).

Recovery has been defined as the process of gaining a new meaning and purpose in life by addressing the challenges associated with mental disorders (Chiba et al., 2014). Mental recovery is considered a process rather than an outcome. Acquiring a profession, working at a job, pursuing an academic life, performing routine tasks, and adapting to ordinary life significantly contribute to recovery (Drake and Whitley, 2014). Efforts to cope with one’s disease, setting personal goals and making progress toward achieving them are significant steps that can lead to mental recovery (Çam and Engin, 2014; Schrank and Slade, 2007; Yaman and Yılmaz, 2020).

Hope, responsibility, self-esteem, goal-setting, assuming social roles, and managing symptoms and stigma are considered essential components of recovery (Schrank and Slade, 2007). Accepting one’s disorder and symptoms and moving on with one’s life allows the individual to experience a sense of recovery (Öztürk et al., 2021). The affects of stigma on self-esteem also negatively affect mental recovery (Lahera et al., 2018). Proper management of the recovery process keeps the individual’s disability at a minimum, improves their adaptation to daily life and makes it possible for the person to live in a more healthy way (Çam and Engin, 2014; Liberman, 2008; Uzun et al., 2018).

In the light of the information mentioned above, disability, internalized stigma, and mental recovery are three important concepts that affect the quality of life of individuals with BD. While disability refers to the loss of functionality due to the symptoms of the illness, internalized stigma occurs when individuals accept and internalize social stigma. Internalized stigma can negatively affect individuals’ self-esteem and lead to loss of motivation for treatment. Mental recovery, on the other hand, involves not only relief from symptoms but also building a meaningful life and returning to social roles. These concepts interact with each other; internalized stigma can increase disability, while spiritual recovery can reduce disability and stigma. The recovery process can balance the negative interaction between these three concepts by increasing self-esteem and social inclusion.

## 2 Current study and research hypotheses

This study primarily aimed to examine the correlation between disability, internalized stigma and mental recovery of patients with BD. It further determines how internalized stigma and disability predict mental recovery.

The purpose of nursing care provided to patients with BD is to ensure the patient’s safety, support the patient in regaining their prior social life, and maintain the recovery (Temel and Kutlu, 2019). The patient should be evaluated holistically while providing nursing care (Akbaş and Yiğitoğlu, 2020). Individuals with BD spend 20% of their lives in hospital (Angst and Sellaro, 2000), and the recurrence rate within the first 2 years is 60% (Yeloğlu, 2017; Yeloğlu et al., 2021). These relapses can cause cognitive and functional disruptions, which eventually result in a disability. It is therefore important, for treatment and care, to focus on ensuring the recovery of patients, improving their well\-being and reintegrating them into society (Ünsal et al., 2014). In the current study, we first examined whether there were differences between internalized stigma and the recovery process based on the level of disability. Furthermore, we assessed the affects of disability and internalized stigma of mental illness on the recovery process. The research hypotheses were as follows:

*H_1_* = *There are differences between internalized stigma and the recovery process based on the level of disability.*

*H_2_* = *Disability and internalized stigma of mental illness predict the recovery process.*

The results obtained from the study will facilitate the management of the symptoms and self-care in both the patients and their families and friends throughout the disease and remission during and after hospitalization. They will provide a guide on how to increase quality of life and functionality.

## 3 Materials and methods

### 3.1 Study design and recruitment

This study was planned as a descriptive cross-sectional study. Patients between 18 and 65 years of age who were registered with Community Mental Health Centers (CMHCs) in two different provinces and diagnosed with BD in remission were included in the study. A total of 103 patients participated in the study, which was conducted between November 2021 and November 2022.

### 3.2 Data collection and inclusion criteria

The researcher visited the CMHCs, obtained the consent of the patients who met the inclusion criteria, and administered the survey after interviewing them in a suitable office. To participate in this study, the participants had to meet the following criteria: Being between 18 and 65 years of age, being followed up with a diagnosis of BD in the CMHC, being able to communicate verbally, having a sufficient cognitive level to answer the questions and not having been co-diagnosed with another psychiatric disorder.

### 3.3 Participants

G*Power 3.1.9.7 was used for the power analysis of the study with 103 participants. The results of the analysis performed to determine the correlation between the scores for the Internalized Stigma of Mental Illness (ISMI) Scale and the Recovery Process Inventory (RPI) revealed that Type 1 error = 0.05, affect size = 0.8657 and power = 1.00 for *n* = 103 patients. Accordingly, the study’s sample size was deemed sufficient and the study was completed with 103 participants.

Forty-seven patients from Bolu CMHC and 56 patients from Sakarya CMHC were included in the study. The mean age of the participants was found to be 40.36 ± 10.72; 52.4% were women; 51.5% were married; 27.2% were employed; 41.7% had graduated from high school; 40.8% lived with their parents; 63.1% lived in the city; and 53.4% had an income less than their expenses. While the average duration of the disorder was 13.54 ± 8.68 years, 36.9% of them had a family history of mental disorder, and 95.1% regularly used the recommended treatment (Table 1).

**TABLE 1:** Sociodemographic characteristics.

Variable	Sub-variable	n/$\bar{\text{x}}$ ± σ	%/min–max
Community Mental Health Center	Sakarya	56	54.4
	Bolu	47	45.6
Age		40.36 ± 10.72	19–64
Gender	Female	54	52.4
	Male	49	47.6
Marital status	Married	53	51.5
	Single	50	48.5
Employment status	Employed	28	27.2
	Unemployed	75	72.8
Educational status	Elementary school	41	39.8
	High school	43	41.7
	University	19	18.4
Person(s) living together	Alone	7	6.8
	Only spouse	15	14.6
	Spouse and children	36	35.0
	Parents	42	40.8
	Flat-mate	3	2.9
Place of residence	Village	14	13.6
	District/town	24	23.3
	City	65	63.1
Perceived income	Income less than the expenses	55	53.4
	Income equal to the expenses	48	46.6
Average duration of the disorder		13.54 ± 8.68	1–49
Family history of mental disorder	Yes	38	36.9
	No	65	63.1
Regular use of the recommended treatment	Yes	98	95.1
	No	5	4.9

N, number; $\bar{\text{x}}$, arithmetic mean; σ, standard deviation; %, percentage.

### 3.4 Measures

#### 3.4.1 Sociodemographic data

These data were collected using a survey form designed by the researchers. This included questions about age, gender, marital status, employment status, educational status, person(s) living together, place of residence, perceived income, duration of the disorder, mental disorder in their family history and regular use of the recommended treatment.

#### 3.4.2 Short disability assessment schedule (DAS-S)

This measurement was developed by Stewart et al. (1988) to evaluate physical and social disability (e.g., *Your personal problems affect your productivity at home, at school or at work has it reduced?*) (Tel et al., 2014). The mean score obtained from the DAS-S, which consists of 11 questions, ranges between 0 and 22. A score between 0 and 4 is evaluated as “no disability,” between 5 and 7 is evaluated as “mild disability,” between 8 and 12 is evaluated as “moderate disability,” and between 13 and above it is evaluated as “severe disability” (Tel et al., 2014). The validity and reliability of the Turkish version of the survey were confirmed by Kaplan (1995). The Cronbach’s alpha value in this study was calculated as 0.92.

#### 3.4.3 Internalized stigma of mental illness (ISMI) scale

This is a four-point Likert-type self-report scale consisting of 29 items aiming to assess the level of internal stigma (e.g., *I feel out of place in the world because I have a mental illness*) (Ritsher et al., 2003). The scale consists of five sub-scales: alienation, endorsement of stereotypes, perceived discrimination, social withdrawal and resistance to stigma. The total ISMI Scale score, calculated by adding the five sub-scales scores, varies between 4 and 91. Higher scores on the ISMI scale indicate that the individual experiences severe internalized stigma in a negative dimension. The validity and reliability of the Turkish version of the scale were confirmed by Ersoy and Varan (2007). The Cronbach’s alpha values in this study were found to vary between 0.63 and 0.92.

#### 3.4.4 Recovery process inventory (RPI)

The RPI consists of 22 items (e.g., *Even when I don’t care about myself, other people do*) and six subscales: anguish, connected to others, confidence and purpose, others’ care/help, living situation, and hopeful/cares for self (Jerrell et al., 2006). It is a five-point Likert-type scale, and the total score that can be obtained varies between 22 and 110. A lower score for the “anguish” subscale represents a negative view of recovery, while lower scores for the other subscales represent a more positive view of the individual’s recovery. The total RPI score is calculated by adding the scores for the six subscales. A lower total score from the scale is considered to indicates a positive recovery process. The validity and reliability of the Turkish version of the scale were confirmed by Yalçıner et al. (2019). The Cronbach’s alpha values in this study were found to vary between 0.67 and 0.94.

### 3.5 Data analysis

Statistical analyses were performed using SPSS 26.0 at a significance level of *p* ≤ 0.05. The data analyzed were presented as numbers, percentages, mean, minimum and maximum values and standard deviation. Whether there was a significant difference between the variables was tested by testing the significance of the difference between the means of two variables (independent *t*-test) for the groups of two and by one-way ANOVA for groups of more than two variables. At the end of the analysis, homogeneity of variance was tested using the Levene test. Then the multiple comparison test (Bonferroni or Tamhane’s T^2^) was applied to find out which group or groups caused the difference. Pearson correlation analysis was used to examine the relationship between numerical measurements, and multiple linear regression analysis was used to examine the factors affecting the RPI. The reliability of the scales was confirmed using the Cronbach’s alpha value.

## 4 Results

### 4.1 Preliminary analysis

It was determined that 33% of the participants reported not having disability, 25.2% had mild disability, 30.1% had moderate disability, and 11.7% had severe disability. The mean scores for the DAS-S, the ISMI Scale and the RPI were 7.13 ± 4.91, 74.89 ± 11.30, and 65.74 ± 12.67, respectively (Table 2).

**TABLE 2:** Mean and standard deviation values of, and relationship between, the ISMI scale, RPI and DAS-S.

Scale	$\bar{\text{x}}$ ± σ	Min–max	1	2	3	4	5	6	7	8
DAS-S	7.13 ± 4.91	0–21	1	0.337*	0.389*	0.255*	0.364*	0.316*	0.097	0.195*
ISMI scale	74.89 ± 11.30	41–94	–	1	0.879*	0.856*	0.754*	0.857*	0.811*	0.865*
Alienation	16.67 ± 2.74	7–22	–	–	1	0.635*	0.624*	0.761*	0.632*	0.706*
Endorsement of stereotypes	15.49 ± 2.92	9–24	–	–	–	1	0.561*	0.638*	0.701*	0.768*
Perceived discrimination	13.50 ± 2.15	6–19	–	–	–	–	1	0.575*	0.479*	0.632*
Social withdrawal	16.69 ± 3.02	7–22	–	–	–	–	–	1	0.549*	0.701*
Resistance to stigma	12.55 ± 2.69	8–18	–	–	–	–	–	–	1	0.789*
RPI	65.74 ± 12.67	28–86	–	–	–	–	–	–	–	1
DAS-S	*n*	%	–	–	–	–	–	–	–	–
No disability	34	33.0	–	–	–	–	–	–	–	–
Mild disability	26	25.2	–	–	–	–	–	–	–	–
Moderate disability	31	30.1	–	–	–	–	–	–	–	–
Severe disability	12	11.7	–	–	–	–	–	–	–	–

$\bar{\text{x}}$, arithmetic mean; σ, standard deviation; r, pearson correlation coefficient; ISMI scale, internalized stigma of mental illness scale; RPI, recovery process inventory; DAS-S, short disability assessment schedule,

*p < 0.05.

The correlations between the scores for the DAS-S, ISMI Scale and RPI were examined in this study. The results of the analyses showed that there were significant positive correlations between DAS-S and ISMI Scale (r: 0.337, *p* < 0.05) and its subscales alienation (r: 0.389, *p* < 0.05), endorsement of stereotypes (r: 0.255, *p* < 0.05), perceived discrimination (r: 0.364, *p* < 0.05), and social withdrawal (r: 0.316, *p* < 0.05), while there was no significant relationship with resistance to stigma (r: 0.097, *p* > 0.05). There were a significant correlations between RPI and DAS-S and ISMI Scale and all its subscales (*p* < 0.05) (Table 2).

### 4.2 Supplementary analysis

The DAS-S scores indicated that there was no significant difference between the resistance to stigma (F: 0.175, p: 0.913) and endorsement of stereotypes (F: 2.067, p: 0.109) subscales of the ISMI Scale; however, there was a significant difference between the ISMI Scale total (F: 3.473, p: 0.019), perceived discrimination (F: 7.041, p: 0.001), alienation (F: 4.239, p: 0.007), and social withdrawal (F: 3.886, p: 0.011) subscales of ISMI Scale (Table 3).

**TABLE 3:** Comparison of ISMI scale and RPI scores based on DAS-S score groups.

Scale		DAS-S				F	*P*
		**No disability**	**Mild disability**	**Moderate disability**	**Severe disability**		
		**$\bar{\text{x}}$ ± σ**	**$\bar{\text{x}}$ ± σ**	**$\bar{\text{x}}$ ± σ**	**$\bar{\text{x}}$ ± σ**		
ISMI scale	Total	70.26 ± 13.88^b^	77.08 ± 8.92	76.06 ± 10.04	80.25 ± 6.08^a^	3.473	0.019*
	Alienation	15.50 ± 3.39^b^	16.92 ± 2.24	17.10 ± 2.23	18.33 ± 1.50^a^	4.239	0.007*
	Endorsement of stereotypes	14.79 ± 3.51	15.96 ± 2.37	15.26 ± 2.58	17.00 ± 2.52	2.067	0.109
	Perceived discrimination	12.24 ± 2.54^b^	14.35 ± 1.57^a^	13.94 ± 1.81^a^	14.08 ± 1.24^a^	7.041	0.001*
	Social withdrawal	15.38 ± 3.34^b^	17.00 ± 2.32	17.29 ± 3.10	18.17 ± 1.95^a^	3.886	0.011*
	Resistance to stigma	12.35 ± 2.98	12.85 ± 2.89	12.48 ± 2.64	12.67 ± 1.37	0.175	0.913
RPI	RPI	61.56 ± 13.29	70.00 ± 12.52	65.55 ± 12.36	68.83 ± 8.90	2.568	0.059
	Anguish	25.21 ± 5.74^b^	28.42 ± 4.63^a^	26.45 ± 4.27	29.08 ± 3.48^a^	3.165	0.028*
	Connected to others	7.59 ± 2.16	9.08 ± 2.38	8.45 ± 2.67	8.92 ± 1.68	2.292	0.083
	Confidence and purpose	10.65 ± 2.63	12.12 ± 2.44	11.55 ± 2.77	12.25 ± 2.30	2.054	0.111
	Others’ care/help	8.32 ± 2.03	9.38 ± 2.04	9.16 ± 1.85	8.08 ± 1.51	2.411	0.071
	Living situation	4.85 ± 1.31	5.27 ± 1.40	4.58 ± 0.92	4.83 ± 0.83	1.611	0.192
	Hopeful/cares for self	4.94 ± 1.54	5.73 ± 1.43	5.35 ± 1.36	5.67 ± 1.56	1.669	0.179

^a,b^The mean differences between groups (*^a^*highest mean). F, one-way ANOVA test.

*p < 0.05.

$\bar{\text{x}}$, arithmetic mean; σ, standard deviation; ISMI scale, internalized stigma of mental illness scale; RPI, recovery process inventory; DAS-S, short disability assessment schedule.

Next, the scores obtained from the RPI subscales were compared according to the disability levels obtained from the DAS-S scores. The analysis results indicated no statistically significant difference between the RPI total, connected to others, confidence and purpose, others’ care/help, living situation and hopeful/care for self-subscales of the RPI (*p* > 0.05). On the other hand, it was concluded that the difference between DAS-S and the anguish subscale of the RPI was statistically significant (F: 3.165, p: 0.028) (Table 3).

Factors affecting the recovery process were examined by applying multiple linear regression analysis. The analysis results show that the model is generally significant (F = 43.343, *p* < 0.001, R^2^: 0.787). Among the independent variables examined in the analysis, endorsement of stereotypes (B = 1.012, *p* = 0.003), social withdrawal (B = 0.852, *p* = 0.011) and resistance to stigma (B = 1.916, *p* = 0.001) show a significant and positive affect. However, other variables, especially alienation (B = 0.286, *p* = 0.471), perceived discrimination (B = 0.759, *p* = 0.062) and different levels of disability, do not have a statistically significant affect (*p* > 0.05) (Table 4).

**TABLE 4:** Examining the factors affecting RPI.

Independent variable		Unstandardized coefficient		Standardized coefficient	t	P	95.0% confidence interval		VIF
		**B**	**Std. deviation**	**Beta**			**Lower limit**	**Upper limit**	
	(Constant)	−3,903	4,350	–	−0,897	0,372	−12,540	4,734	–
DAS-S	Mild disability/no disability	2,927	1,739	0,101	1,684	0,096	−0,525	6,380	1,582
	Moderate disability/no disability	−0,104	1,665	−0,004	−0,062	0,950	−3,410	3,203	1,617
	Severe disability/no disability	−0,144	2,254	−0,004	−0,064	0,949	−4,619	4,330	1,449
ISMI scale	Alienation	0,286	0,395	0,062	0,724	0,471	−0,498	1,070	3,210
	Endorsement of stereotypes	1,012	0,336	0,233	3,008	0,003*	0,344	1,680	2,648
	Perceived discrimination	0,759	0,402	0,129	1,888	0,062	−0,039	1,557	2,052
	Social withdrawal	0,852	0,329	0,203	2,588	0,011*	0,198	1,506	2,714
	Resistance to stigma	1,916	0,349	0,407	5,497	0,001*	1,224	2,608	2,411

F: 43.343, *p* < 0.001, R^2^: 0.787, ISMI scale, internalized stigma of mental illness scale, RPI, recovery process inventory, DAS-S, short disability assessment schedule.

*p < 0.05.

## 5 Discussion

The present study’s findings provided important data on the distribution of the disability levels of the participants. One-third of the participants had no disability, one-quarter had mild disability, about one-third had a moderate disability, and more than one-tenth had severe disability. These findings are important for understanding the different affects of bipolar illness (BD) on the lives of individuals. For example, Saka et al. (2001) found that 40%–50% of individuals with BD had mild, moderate and severe disability. In a study conducted by Guzzo et al. (2022) to evaluate functionality in BD, it was concluded that functionality was slightly impaired. The findings of Candan (2019) support our study findings, but differences are also observed. These differences may result from the clinical course of BD, individual and environmental factors, and treatment approaches (Lee et al., 2015; Guzzo et al., 2022). The frequency of attacks in BD makes it challenging to comply with treatment and causes a loss of functionality in occupational, social and academic areas. Loss of functionality affects the level of disability of patients. In this context, the fact that the disability levels of the patients in our sample group were found to be compatible with some studies but different from the results of others can be explained by differences in the course of the disease and the care and treatment applied (Öztürk et al., 2021). Although disability occurs at different levels, it is a common outcome in patients diagnosed with bipolar disorder. The level of disability can be alleviated with psychosocial support, cognitive behavioral therapies and psychoeducation provided to patients and their families (Öztürk et al., 2021).

The importance of internalized stigma in BD is also emphasized. The mean ISMI Scale score in our study was calculated as 74.89 ± 11.30. The literature review indicated that mean ISMI Scale score in studies conducted with patients with mental disorders varies between 36.18 ± 10.75 and 60.40 ± 9.60 (Çalışkan, İlter et al., 2023; de Filippis et al., 2022; Hançer et al., 2020; Türk and Bulut Uğurlu, 2023; Çam and Çuhadar, 2011; Kurnaz, 2019). Brohan et al. (2011) concluded that internalized stigma is observed in one in five people diagnosed with BD. Stigma toward individuals with BD increases the person’s social isolation and makes compliance with treatment difficult (de Filippis et al., 2022). Those living in rural areas and small settlements may be exposed to a greater degree of stigma and more restrictions in their social relationships due to the course of the disorder, as their relationships with others are often close and intricate. Accordingly, the higher ISMI Scale scores of the participants in the study sample may be attributed to the fact that a significant portion of the participants lived in rural areas, were unemployed and had limited social relations. The supportive social structure in the community is believed to contribute to the patient’s understanding of their symptoms and whether they can gain skills to cope with the disorder. The search for meaning in life further contributes to mental recovery. The fact that most of the participants in the current study lived with their families suggests that their social support is sufficient and their recovery process is positive.

The potential interactions between disability, internalized stigma and psychological recovery in BD are important. Our findings showed that participants experienced internalized stigma and disability in the last month, which in turn affected their recovery process. Grover et al. (2016) in India found that internalized stigma was associated with prolonged depressive episodes and increased prevalence of disability. Individuals who have experienced internalized stigma often have severe health conditions and are more likely to be hospitalized. There is a vicious cycle between internalized stigma and disability (Aydemir, 2013). Our results provide important insights into how internalized stigma affects individuals’ social and psychological functioning by linking significant positive correlations between the DAS-S and the ISMI Scale, particularly with the subscales of alienation, stereotype confirmation and social withdrawal. The critical implications of potential interactions between internalized stigma and disability on psychological recovery processes have long been highlighted in the literature (Jahn et al., 2020). In this context, findings suggest that disability is associated with dimensions of stigmatization, such as alienation and social withdrawal, which may cause individuals to withdraw from their social environment and face difficulties in recovery processes. Grover et al. (2016) also reported that internalized stigma is associated with prolonged depressive episodes and increased prevalence of disability. This supports the findings of our study. In particular, the relationships observed with sub-dimensions such as disability and recovery process, alienation and perceived discrimination lead individuals to develop negative perceptions toward themselves and to decrease their interactions with their social environment. Aydemir (2013) drew attention to the vicious circle between internalized stigma and disability and stated that this situation reinforces the stigmatization experiences of individuals and negatively affects the recovery process. Similarly, our study’s significant correlation between the recovery process and alienation and social withdrawal reveals how this reinforcing cycle affects individuals’ social participation and recovery process. In summary, the findings of our study show that the interaction of internalized stigma with sub-dimensions of disability and recovery process, such as perceived discrimination, social withdrawal and alienation, constitutes an important obstacle in coping with mental disorders. In line with similar studies, it can be said that this situation negatively affects individuals’ social and psychological functioning, so it is important to develop strategies to reduce stigmatization.

Similarly, Temesgen et al. (2019) found that the disability experienced by individuals with serious psychiatric disorders decreased as they recovered from their illnesses. The recovery among patients with BD improves their skills to understand and make sense of their disease and facilitates a freer and more satisfactory life, thus reducing the affect of disability (Öztürk et al., 2021). In another study, İpçi et al. (2020) found a positive correlation between subjective recovery and functioning, suggesting that recovery processes are associated with increased participation in life and reduced disability. Interestingly, individuals with moderate or severe disabilities who experienced significant distress were found to have a more positive trajectory of recovery compared to their non-disabled counterparts. This counterintuitive result may be attributed to the fact that as the level of disability increases, the support received from family members and health organizations increases.

Our results also provide important findings by focusing on the factors that are affective in the mental recovery process. The research results show that resistance to stigmatization and confirmation of stereotypes contribute significantly and positively to healing. This situation indicates that individuals’ efforts to cope with social prejudices and reconstruct these prejudices support their mental recovery. In line with the literature, the studies also reveal that stigmatization negatively affects mental recovery and that resistance mechanisms developed against stigmatization play a critical role in this process (Chang et al., 2016; Oexle et al., 2018). Resistance to stigmatization supports mental wellbeing and social functionality by increasing individuals’ self-esteem and independence. Öztürk et al. (2021) also stated that as the patients’ sense of recovery increases, the functionality of individuals increases, and the perceived severity of disability negatively affects this process. However, the lack of a significant affect of alienation and different disability levels on the recovery process in this study suggests that these factors may be associated with more indirect or context-specific affects. In conclusion, this study shows that developing resistance to stigmatization and combating social prejudices support mental recovery. These findings emphasize the importance of interventions targeting the psychosocial empowerment of bipolar patients and guide future studies.

Our study has some limitations. First, the research sample was relatively narrow. We are studying Turkish patients exclusively, which further limits the generalizability of the findings to bipolar patients from different countries and with different cultural characteristics. The specific family structures and relationships within different societies affect the patients’ interactions with their environment. Second, the research data were collected using self-report surveys; this may have improved the relationships between variables but also introduced some bias. Research data should be supported with other types of instruments (e.g., tests evaluated by an expert). Therefore, future studies are needed to investigate this issue using a multi-method approach. Finally, the cross-sectional study design limited the identification and longitudinal examination of causal relationships or the investigation of change across profiles over time. Another limitation of this study is that additional diagnoses and different types of BD (Bipolar I and Bipolar II) were not considered, which may provide a more nuanced understanding of how these factors influence internalized stigma and recovery outcomes.

The findings of this study are important in terms of understanding how patients with bipolar disorder in Turkey develop their relationships with environmental factors. Turkish society has a structure in which family ties are intense and social support is important; this can significantly shape how individuals cope with stigmatization. Therefore, the impact of internalized stigma on mental recovery is of particular importance. Our research aims to raise awareness of this issue and contribute to developing more inclusive treatment approaches for these individuals.

## 6 Conclusion and practical implications

To the best of our knowledge, this is the first study examining the difference between internalized stigma and mental recovery, as well as the affect of internalized stigma and mental recovery on disability, by the level of disability of bipolar patients in remission. The results of this study indicated that the mental recovery of bipolar patients in remission with severe or mild disability was less negatively affected, particularly in terms of the “anguish” subscale, compared to those without disability. It was further observed that disability and internalized stigma affected the recovery process. In conclusion, we hope that this study will provide a framework for further research investigating the level of disability, stigma, and recovery process in all bipolar patients in remission and will hence improve the provision of the care services provided to individuals with chronic mental disorders.

## Data Availability

The raw data supporting the conclusions of this article will be made available by the authors, without undue reservation.
